# A randomised controlled trial to evaluate a medication monitoring system for TB treatment

**DOI:** 10.5588/ijtld.21.0373

**Published:** 2022-01-01

**Authors:** J. Acosta, P. Flores, M. Alarcón, M. Grande-Ortiz, L. Moreno-Exebio, Z. M. Puyen

**Affiliations:** 1 Facultad de Medicina Humana, Universidad Nacional del Centro del Perú, Huancayo, Peru; 2 Instituto Nacional de Salud, Lima, Peru; 3 Escuela de Medicina, Universidad Peruana de Ciencias Aplicadas, Lima, Peru

**Keywords:** dispenser pillbox, digital adherence technologies, treatment adherence, treatment success

## Abstract

**BACKGROUND:**

Adherence to TB treatment and therefore treatment success could be improved using digital adherence technology.

**OBJECTIVE:**

To evaluate the effectiveness of a medication event reminder monitor system (MERM) on treatment success and treatment adherence in patients with drug-susceptible pulmonary TB in Perú.

**METHODS:**

This was an experimental, randomised, open-label, controlled study conducted among patients in the second phase of TB treatment. The intervention group received their medications through MERM with the support of a treatment monitor, whereas the control group used the usual strategy. Participants were followed until they completed the 54 doses of the second phase of treatment.

**RESULTS:**

The study included 53 patients in each group; four in the intervention group withdrew from the study. Treatment success was significantly more frequent in the MERM group (RR 1.15, 95% CI 1.02–1.30; *P* = 0.0322). There was no significant difference in the adherence outcomes; however, the percentage of patients who missed at least one dose and patients with more than 10% of total doses missed were lower in the intervention group.

**CONCLUSION:**

The use of MERM in the second phase of treatment showed a significant improvement in the treatment success rate in patients with drug-susceptible pulmonary TB.

## Abstract (fr)

**CONTEXTE:**

L’observance du traitement antituberculeux et donc la réussite thérapeutique pourraient être améliorées à l’aide d’une technologie numérique dédiée.

**OBJECTIF:**

Évaluer l’efficacité d’un système de surveillance des rappels de prise de médicaments (MERM) sur la réussite thérapeutique et l’observance du traitement chez les patients atteints de TB pulmonaire pharmacosensible au Pérou.

**MÉTHODES:**

Il s’agissait d’une étude contrôlée, ouverte, randomisée et expérimentale réalisée auprès de patients en seconde phase de traitement antituberculeux. Le groupe d’intervention a reçu ses médicaments via le système MERM avec l’aide d’une personne en charge de surveiller le traitement, alors que le groupe témoin a bénéficié de la stratégie habituelle. Les participants ont été suivis jusqu’à ce qu’ils aient reçu les 54 doses de la seconde phase de traitement.

**RÉSULATS:**

L’étude a inclus 53 patients dans chaque groupe ; quatre patients du groupe d’intervention se sont retirés de l’étude. La réussite thérapeutique était significativement plus fréquente dans le groupe MERM (RR 1,15 ; IC 95% 1,02–1,30 ; *P* = 0,0322). Aucune différence n’a été observée en termes d’observance ; toutefois, les pourcentages de patients ayant manqué au moins une dose et de patients avec plus de 10% des doses totales non prises étaient plus faibles dans le groupe d’intervention.

**CONCLUSION:**

L’utilisation du MERM dans la seconde phase du traitement a été associée à une amélioration significative du taux de réussite thérapeutique chez les patients atteints de TB pulmonaire pharmacosensible.

TB is a global public health problem, being the top cause of death from an infectious agent in the world.^1^ As one of the 30 high MDR-TB burden countries, Perú has an incidence rate of 119 per 100,000 population, with an incidence of multidrug- or rifampicin-resistant TB (MDR/RR-TB) of 9.6%.^1^,^2^ In Perú, the treatment for drug-susceptible pulmonary TB (DS-PTB) lasts 6 months and includes two phases supervised by TB programme staff using the directly observed treatment (DOT) strategy.^3^

Duration, complexity and toxicity of the treatment, together with social, economic, patient-related, disease-related and health system-related factors impact adherence to treatment and directly affect the treatment success rate (TSR).^3^–^5^ Non-adherence to TB drugs has an important impact on increasing death rates, treatment failure and cost outcomes, and is a risk factor for the development of MDR-TB.^6^,^7^ In 2018, the TSR in new and relapse cases registered was 85.1%; however, the TSR in patients with MDR-TB is lower (61.4%).^8^

Digital adherence technology (DAT), including phone-based technologies, event monitoring devices, or a combination of these, have been shown to be effective in improving treatment adherence. These facilitate patient-centric approaches for monitoring adherence.^9^–^11^ This study aimed to evaluate the effectiveness of a medication event reminder monitor (MERM) system compared to standard DOT on treatment success and adherence to DS-PTB medication in Perú.

## METHODS

### Study design and population

The study was an experimental, randomised, open-label, controlled study conducted between June 2018 and June 2020 in Callao, Peru, a region with a TB incidence rate of 99.6/100,000 at the beginning of the study.^8^ Participants were recruited from 19 Ministry of Health (MoH) primary healthcare centres (PHCs) with the highest incidence of TB in the region. The participants were patients with DS-PTB, who were at the end of the first phase of the DOT-based treatment for DS-PTB (daily isoniazid [INH], rifampicin [RIF], pyrazinamide [PZA] and ethambutol [EMB] for 2 months) and had a final susceptibility result before inclusion in the study. Participants were adults (age >18 years) who could read and used a mobile phone daily. Patients who were hospitalised, alcoholics or drug addicts, patients without mobile phone network in their homes and patients that TB programme staff considered to be at risk of low adherence to the treatment (due to missed doses in the first phase) were excluded.

Assuming the WHO-recommended TSR of 90%, a TSR of 68% reported in Perú (2013), 95% confidence level and 80% power, with a ratio of 1:1 of control and interventional patients, a sample size of 53 patients for each group was calculated using the formula for comparing two independent proportions. Simple randomisation was performed asking patients who met the selection criteria to choose between two cards (control and intervention group) until the number of participants required for the study in each group was reached regardless of the PHC to which they belonged.

### Treatment administered and evaluations in participants

All participants received thrice weekly INH and RIF during the second phase of treatment. The control group were administered DOT in the PHC by a nurse from the TB programme staff, who supervised treatment administration in the patients’ mouth and recorded it in the TB treatment register as specified in the national guidelines.^3^ The intervention group received their treatment through MERM with the support of treatment monitors, who are health professionals trained to supervise the correct use of MERM, contact the participant to advise on treatment adherence and support the treatment process (clinician evaluation, analysis). All participants were evaluated by the TB programme staff in their PHCs, including a monthly nurse evaluation and analytical evaluation as per the national guidelines.^3^

Patients were followed until they completed the 54 doses of the second phase of treatment (corresponding to 4 months) in order to collect treatment information, and then until the TB programme staff performed a final diagnosis with radiological and microbiology evaluation (usually no more than 1 month). Patients who needed an extension of the 4-month second phase of treatment according to TB programme staff were considered as “clinical failures”.

### Medication Event Reminder Monitor System (MERM)

The MERM used in the study was Wisepill RT2000 (Wisepill Technologies, Cape Town, South Africa); this consisted of an electronic dispenser pillbox, a web server and text messaging.^12^ The MERM used a mobile phone network to monitor patient’s treatment in real-time, sending a signal to a central management system (Wisepill Web Server) every time the dispenser device is opened. The MERM sent a pre-designed short message service (SMS) to the participant’s mobile phone if the device was not opened at the hour scheduled for treatment, and every 30 min until the device was opened with a maximum of three SMS per day. The third SMS was also sent to a previously designated relative and to the treatment monitor who contacted the participant to identify a possible connectivity problem, doses taken before the established hour or to remind the participant to take the dose.

The research group could access the MERM usage reports and SMS sent through the web server. In case of signal transmission malfunction, MERM stored data for later transmission when connectivity was reestablished. However, if the participant opened the device before the scheduled time, the device did not recognise that dose. The device was filled each week in the PHC.

### Outcomes evaluated

The primary outcome measure was treatment success, which included patients with a final diagnosis of “cured” or treatment completed, whereas treatment failure included “person lost to follow-up” or “clinical failure”.^3^

Treatment adherence was measured in terms of the proportion of missed doses, the proportion of patients who missed at least one of the total doses scheduled at the time of inclusion in the study and the proportion of patients who missed >10% of doses.^13^ A missed dose was considered when the patient did not attend the PHC and could not be reached by staff on the day of treatment in the control group, or when the pillbox was not opened on the day of treatment and the treatment monitor had excluded connectivity problems in the MERM group.

### Data analysis

Using the MERM web reports, we identified unscheduled and missed doses and SMSs sent. The reports were exported using MS Excel (Microsoft Office 2016, Redmond, WA, USA) and were modified with the information collected by the treatment monitor, and the epidemiological and clinical information recovered from the medical charts of the participants. The analysis was performed using R software v4.0.2 (R Computing, Vienna, Austria).

For the descriptive analysis, medians, interquartile range (IQR) and percentages were calculated. The relative risk (RR) with 95% confidence intervals (CIs) was reported; the χ^2^, Fisher’s exact test or Wilcoxon test were used to determine the difference between the groups; significance was set at *P* < 0.05.

### Ethics statement and informed consent

The institutional ethics committee of the National Health Institute in Lima, Perú (RD N° 370-2015-OGITT-OPE/INS) approved the study. All patients provided written informed consent before inclusion in the study. Patient information was anonymised and de-identified.

## RESULTS

### Participant characteristics

From June 2018 to February 2020, 159 participants were screened, 112 met the enrolment criteria, and 6 refused to participate. Of the 106 included in the study, 4 from the MERM group were withdrawn: 2 withdrew voluntarily, 1 switched to a different treatment, and the TB programme staff withdrew 1 for suspected misuse of the pillbox. All four were in the MERM group and had participated an average of 70 days in the study. The demographic, social and clinical characteristics of the participants are shown in Table 1.

**Table 1:** Characteristics of participants by treatment strategy used

		DOT (*n* = 53) *n* (%)	MERM (*n* = 49) *n* (%)
Sex	Female	30 (56.6)	23 (46.9)
Age, years	18–35	42 (79.2)	41 (83.7)
	36–59	8 (15.1)	4 (8.2)
	>60	3 (5.7)	4 (8.2)
Highest education grade completed	Primary school	3 (5.7)	2 (4.1)
	Secondary school	31 (58.5)	26 (53.1)
	University	7 (13.2)	11 (22.4)
	Technical school	12 (22.6)	10 (20.4)
District of residence (number of health centres)	Bellavista (*n* = 1)	7 (13.2)	2 (4.1)
	Callao (*n* = 8)	21 (39.6)	16 (32.7)
	Carmen de la Legua (*n* = 2)	1 (1.9)	3 (6.1)
	Mi Perú(*n* = 1)	8 (15.1)	5 (10.2)
	Ventanilla (*n* = 7)	16 (30.2)	23 (46.9)
Employment status	Self-employed	12 (22.6)	19 (38.8)
	Employee	5 (9.4)	3 (6.1)
	Labourer	3 (5.7)	3 (6.1)
	Housekeeper	7 (13.2)	2 (4.1)
	Unpaid housekeeper	5 (9.4)	2 (4.1)
	Unemployed or student	21 (39.6)	20 (40.8)
Income level, PEN	No monetary income	30 (56.6)	25 (51)
	<750	17 (32.1)	14 (28.6)
	750–1500	6 (11.3)	9 (18.4)
	>1500	0	1 (2)
Previous chronic medical condition	Yes	7 (13.2)	12 (24.5)
	No	46 (86.8)	37 (75.5)
Used another medication	Yes	5 (9.4)	7 (14.3)
	No	48 (90.6)	42 (85.7)
Diagnosis at recruitment	New patient	47 (88.7)	45 (91.8)
	Person lost to follow-up	2 (3.8)	0
	Relapse	3 (5.7)	3 (6.1)
	Referred	1 (1.9)	1 (2)
Presentation of treatment provided (INH/RIF)	Single-drug formulations	29 (54.7)	20 (40.8)
	Fixed-dose combination	24 (45.3)	29 (50.2)

DOT = directly observed treatment; MERM = Medication Event Reminder Monitor System; PEN = Peruvian sol; INH = isoniazid; RIF = rifampicin.

Overall, 52.0% of patients were female; the median age was 26.32 years (IQR 21–32.75), 40.2% were unemployed or students, 55.9% had only high school education and 53.9% had no income. Most patients were new and did not use any other medication. Since July 2018, treatment was modified to fixed-dose combination as established by the national TB programme.

### Outcomes and characteristics of treatment

Treatment success was significantly more frequent in MERM group than in the control group, respectively 98.0% and 84.9% (RR 1.15; *P* = 0.0322) (Table 2). Treatment adherence outcomes were not significantly different; however, the proportion of patients who missed at least one dose and patients with more than 10% of total doses missed were higher in the DOT group (Tables 2 and 3). One participant in the control group was lost to follow-up at Month 3 of treatment.

**Table 2:** Treatment and adherence outcomes DOT

		DOT (*n* = 53) *n* (%)	MERM (*n* = 49) *n* (%)	RR (95% CI)	*P* value
Treatment success	Yes	45 (84.9)	48 (98.0)	1.15 (1.02–1.30)	0.0322
	No	8 (15.1)	1 (2.0)		
Patients who missed at least one dose	Yes	15 (28.3)	11 (22.4)	0.79 (0.40–1.56)	0.498
	No	33 (71.7)	38 (77.6)		
Patients with >10% of total doses missed	Yes	7 (13.2)	1 (2.0)	0.15 (0.02–1.21)	0.0613
	No	46 (86.8)	48 (98.0)		

DOT = directly observed treatment; MERM = Medication Event Reminder Monitor System; RR = relative risk; CI = confidence interval.

**Table 3:** Characteristics of treatment of intervention and control group

	DOT				MERM				
	
	Median	IQR	Min	Max	Median	IQR	Min	Max	*P* value
Percentage of doses missed per participant	0	0–1.9	0	33.3	0	0–0	0	15.1	0.310
Total doses received per patient after the first phase of treatment, including doses after the study period	54	54–58	34	177	54	54–55	54	75	0.036

* 20 patients in the DOT group and 11 patients in the MERM group.

DOT = directly observed treatment; MERM = Medication Event Reminder Monitor System; IQR = interquartile range; min = minimum; max = maximum.

Although the analysis was performed according to per protocol (PP) principle, we also re-analysed treatment success with the four patients who withdrew from the study, considering all four as unsuccessful treatment, and the RR was 1.09 (95% CI 0.94–1.27; *P* = 0.3903). Patients in both groups had their treatment extended (more than 54 doses) due to delays in medical discharge; however, the extended treatment period was not considered in the analysis of treatment adherence. In order to evaluate the final treatment received for the participants, we calculated the total number of doses received by each patient in each group after the first phase of the study. This was found to be significantly higher in the control group (Table 3).

### Unscheduled doses and connectivity problems detected in the MERM group

Of the 49 participants in the MERM group, all had at least one episode of doses after the scheduled hour, with a total of 586 (22.6%) episodes of 2,596 possible. Four patients had episodes of doses after the scheduled hour without an SMS sent by the MERM (1–6 episodes per patient) and five patients had episodes of missed doses without any SMS sent. In 37 (75.5%) participants, an SMS was sent at least in one episode, although the device was opened at the scheduled hour. Most of these patients lived in the districts of Callao (32.4%) and Ventanilla (40.5%). In 24 (49.0%) participants, a reminder SMS was sent in at least one episode because the device was opened before the scheduled hour. The proportion of missed doses out of the possible identified (doses after the scheduled hour plus missed doses) was 4.1%. Characteristics of doses taken and SMS received are given in Table 4.

**Table 4:** Description of doses taken according to the hour scheduled and SMS sent in the group using the Medication Event Reminder Monitor System

	Minimum	Maximum	Median	IQR
Doses per participant after the scheduled hour who received SMS	2	35	11	10–16
Doses per participant after the scheduled hour who received only 1 or 2 SMS	1	25	8	3–11
Doses per participant before the hour scheduled	0	17	0	0–2
Doses per participant with an SMS sent due to connectivity problems (drug intake was at the scheduled hour)	0	16	2	1–6

SMS = short message service; IQR = interquartile ranges.

## DISCUSSION

This study evaluated the effectiveness of a MERM system on treatment success and adherence in patients with DS-PTB in a high TB incidence region in Perú, and found that the probability of treatment success with MERM strategy was 1.15 times than that with the standard DOT strategy (*P* = 0.032). Although there was no significant difference in the principal adherence outcomes, the percentage of doses missed, the proportion of patients who missed at least one dose or missed more than 10% of doses was higher in the DOT group.

The WHO has recommended the use of DAT, including MERM, SMS/mobile phone texting and video-supported directly observed therapy (vDOT), to improve adherence to TB treatment.^14^ The Stop TB Partnership’s Global Drug Facility (GDF) recently included the first MERM in the GDF’s product catalogue, specifically the Wisepill evriMED smart medication container.^15^

Studies with MERM show promising results in patients with DS-PTB in improving treatment success,^16^–^18^ as observed in our study, where the probability of treatment success was higher with MERM strategy. However, an analysis including the four patients with an unsuccessful treatment outcome who were withdrawn from the MERM group showed no significant difference (RR 1.09, 95% CI 0.94–1.27; *P* = 0.3903). In line with these results, the adherence outcomes show no difference between the two groups in contrast with previous studies where MERM improves adherence outcomes.^17^–^19^ These results may have been due to the restricted selection criteria used in this study, where we excluded patients at risk of low adherence or at risk of being lost to follow-up.

The total number of doses received per patient after the first phase of treatment, including doses after the study period, were significantly higher in the DOT group than in the MERM group (*P* = 0.036; Table 3). This difference is explained by the lower TSR in the control group, which led to an extension of treatment, but also because, as described in a previous study,^19^ the treatment monitor in the MERM group were in close communication with the patients, and was able to identify problems in the clinical follow-up at the PHC, leading to a rapid final diagnosis after the 54 doses of the second phase of treatment.

A recent study under programme settings in China reported fewer deaths in the patients who started treatment with MERM but found no significant difference (2.5% vs. 3.5%; *P* = 0.191).^20^ These results confirm the opinion of researchers who highlight the importance of conducting better-quality studies to determine the impact of DAT in national TB programmes as evidence of the effect of DAT in improving TB care remains limited.^11^,^21^

There is evidence of acceptability of and satisfaction with MERM as a treatment strategy for DS-PTB and HIVamong patients and health personnel.^19^,^22^–^26^ Also, it has been reported that the use of MERM significantly reduces the workload of health professionals.^19^ In our study, only two participants in the MERM group withdrew from the study at Months 1 and 2 since inclusion, in contrast with that observed in other studies where withdrawal was reported in the last 2 months of treatment.^19^

Before MERM can be implemented, its effectiveness and acceptability in each population should be ascertained. As our study included participants with a low probability of being lost to follow-up, the results cannot be extrapolated to the general population. A study under a programme setting found that children (<15 years), the elderly (>65 years), semi-skilled or unemployed people, people with TB pleurisy and previous TB treatment were less likely to use MERM within the first month.^27^

Also, it is essential to consider an appropriate mobile phone network because problems in transmission of the open device signal could result in SMS being sent when it is not required.^22^ In this study, 75.5% of the participants had at least one episode in which an SMS was sent by mistake due to connectivity problems, and 10.2% of the participants had episodes of missed doses without any SMS sent (seven episodes in total). This last group could have taken their dose later in the day if they had received the SMS.

Finally, for correct implementation, it is crucial to use MERM adherence data to support an appropriate patient-centric approach to improve adherence behaviour, address its causes, or switch to DOT when necessary.^11^,^13^,^23^,^28^ In our study, only one patient in the MERM group was withdrawn due to suspicion of low adherence and started on DOT on the suggestion of TB programme staff.

One problem identified using MERM is the possibility of removal of the medication from the pillbox, for example, for work-related reasons, which prevents the recording of pill dispensing.^11^,^23^ In this scenario, there is a possibility that some of the 196 episodes reported in our study as connectivity problems could be the consequence of the previous removal of the medication from the pillbox; this would explain the higher rate of treatment success in the MERM group even though there was no significant difference in adherence outcomes when compared to the DOT group. It is, therefore, necessary to carefully plan the time for medication as per the patient’s daily routine.

In order to certify the use of treatment in the MERM group, we determined monthly concentrations of INH and RIF in plasma only in these patients without prior notification. The median concentration level was respectively 5.3 μg/mL (IQR 3.40–7.82)) and 5.7 μg/mL (IQR 8.20–2.18)) for INH and RIF; however, respectively 25 and 5 of 196 determinations had undetectable RIF and INH levels (corresponding to 16 patients). All these patients had a successful treatment outcome; therefore, the undetectable levels may have been because the time for blood sampling had not been standardised and the analysis may have been performed after the peak hour. However, these observations will require further analysis to determine if the pharmacokinetics of RIF or INH is different in Peruvians as proposed previously by Requena-Mendez et al.^29^ The patient who was withdrawn from the study by the TB programme staff had three of four RIF and INH plasma concentration analyses with undetectable values of zero.

Limitations of this study include the population selected, who had a low probability of being lost to follow-up and low risk of poor adherence to treatment; therefore, our results cannot be used to predict the effectiveness of MERM in other populations. Measurement bias was detected in case connectivity problems in the MERM group; furthermore, we assumed that a dose had been taken when the pillbox was opened, although this may not be true. These problems were resolved by the inclusion of a treatment monitor, who reduced the risk of errors in the treatment registry in the MERM group. The study did not assess the acceptability of the MERM system in patients or health professionals, although several studies report high rates of acceptability in resource-limited settings.

To our knowledge, this is the first Latin American study on MERM technology in TB patients. The use of MERM led to a significant improvement in TSR and adherence outcomes in patients with DS-PTB. Although the significant difference in treatment success is lost when we included the four patients withdrawn from the study, our results show that MERM could be used as a substitute for DOT strategy to improve the treatment of patients with TB in Latin America.
